# Deepening the Understanding of Inflammatory Conditions Through Rheumatology Education in Family Medicine: An Autoethnography

**DOI:** 10.7759/cureus.67741

**Published:** 2024-08-25

**Authors:** Ryuichi Ohta, Chiaki Sano

**Affiliations:** 1 Community Care, Unnan City Hospital, Unnan, JPN; 2 Community Medicine Management, Faculty of Medicine, Shimane University, Izumo, JPN

**Keywords:** primary care, comprehensiveness, uncertainty, continuity, autoethnography, medical education, rural health, family medicine, psoriatic arthritis, rheumatoid arthritis

## Abstract

Introduction

Rheumatic diseases, such as rheumatoid arthritis (RA) and psoriatic arthritis, significantly affect quality of life and require comprehensive management, especially where rheumatologists are scarce. Family physicians in rural settings play a crucial role in providing this care. This study aims to elucidate the educational process for family physicians in managing RA within rural hospitals.

Methods

Using a qualitative autoethnographic approach, we explored the learning experiences of family physicians at Unnan City Hospital in rural Japan. Data were collected through semi-structured interviews, direct observation, reflective field notes, and informal conversations with participants. The study focused on the practical education of RA management facilitated by a family physician with expertise in rheumatology.

Results

Three main themes emerged: (1) comprehending arthritis as a systemic disease, (2) managing dynamic conditions with prudence, and (3) providing comprehensive and continuous care amid uncertainty. Participants initially viewed arthritis as a localized condition but learned to approach it as part of systemic inflammation. They recognized the fluctuating nature of autoimmune diseases, emphasizing the need for cautious and flexible management. Continuous monitoring and a comprehensive approach to patient care with enduring uncertainty were identified as essential for effective RA management.

Conclusion

The study highlights the importance of experiential learning and mentorship in educating family physicians about RA in rural settings. Understanding arthritis as a systemic condition, exercising prudence in treatment, and maintaining comprehensive care amid uncertainty are crucial components of effective management. These findings inform the development of targeted educational programs for family medicine residents, ultimately enhancing patient care in rural areas. Future research should include multiple rural settings and quantitative data to validate and expand upon these insights.

## Introduction

Rheumatic diseases, particularly rheumatoid arthritis (RA) and psoriatic arthritis, are prevalent conditions that significantly impact the quality of life of those affected [1,2]. Family physicians often find themselves at the forefront of managing these conditions comprehensively, especially in settings where rheumatologists are scarce [3]. This shortage of specialized rheumatology care drives the rising demand for family physicians to manage rheumatic diseases [4]. Effective management in primary care, coupled with strategic collaboration with rheumatologists, is essential to provide comprehensive care for patients with rheumatic diseases [5].

Family physicians' education on rheumatic diseases varies widely and must encompass a broad spectrum of knowledge [6]. This includes specific rheumatic diseases, associated infections, and other inflammatory conditions [7]. A deep understanding of inflammatory processes is crucial for family physicians to accurately rule out critical diseases and tailor effective treatment plans for individual patients [7]. This comprehensive approach to patient care underscores the necessity for an in-depth education that integrates the pathophysiology of inflammation with practical clinical skills.

The educational journey for family physicians to understand inflammatory conditions requires continuous learning and active discussion among healthcare providers. Delving into the complexities of immunology and the pathophysiology of these diseases is essential to enhancing the quality of care provided [8]. However, this learning process can be intricate, influenced by the diverse characteristics of both learners and educators [8]. By clarifying the educational process, we can improve the teaching and learning of rheumatology in primary care, particularly in rural settings where resources are often limited.

The incidences of RA, polymyalgia rheumatica, and other rheumatic diseases have notably increased among the aging population, necessitating effective management by family physicians [9]. This trend is particularly pronounced in rural areas, where the aging population is growing rapidly. Consequently, there is an urgent need for comprehensive care for older patients with RA in these contexts [10]. Enhancing the education of family physicians regarding the management of RA is vital to meet this demand. Clarifying the educational process specific to rural contexts can significantly benefit the quality of care for patients with RA.

In rural areas, the shortage of rheumatologists exacerbates the challenge of providing adequate education on rheumatic diseases. As a result, education often falls to a limited number of rheumatologists and family physicians with a particular interest in rheumatology [4]. This research aims to clarify the learning process for family physicians regarding RA in rural hospitals. By focusing on the education provided by family physicians with a keen interest in rheumatology, this study seeks to promote effective RA management in rural settings, ultimately enhancing patient outcomes.

## Materials and methods

Research design

This study utilized a qualitative research approach, specifically autoethnography, to explore how family physicians learn about RA within the context of education provided by the Family Medicine Department at a rural community hospital. The perspective of the department's director, combined with critical reflections among team members, provided a unique lens for this examination [11]. Autoethnography facilitates a deep exploration of rheumatology education from an insider's viewpoint, offering detailed insights into the practical aspects of teaching RA in rural and community hospitals, which is valuable for medical educators [12]. This approach is efficient for analyzing healthcare environments' intricate social interactions and educational processes. To address potential limitations, the research design incorporated a combination of one-on-one semi-structured interviews, observations of the researcher's and participants' interactions and emotions, detailed field notes on the teaching and learning activities of medical teachers and residents, and reflective discussions with participants to validate findings through member checking [11].

Setting

The study took place at Unnan City Hospital, located in rural southeastern Shimane, Japan. This hospital has 281 care beds, including 160 for acute care, 43 for comprehensive care, 30 for rehabilitation, and 48 for chronic care. It serves as a training facility for family medicine, offering residents a comprehensive educational experience across various clinical settings, such as inpatient, outpatient, home, and community care. The Family Medicine Department oversees the educational curriculum, emphasizing interprofessional collaboration with other healthcare professionals, including dentists, pharmacists, therapists, nurses, and nutritionists. This collaborative approach has reduced readmission rates among older inpatients, fostering interprofessional education among residents [13,14].

Unnan City Hospital also provides community-based medical education to students and residents from various institutions, offering a broad range of clinical experiences across different care settings. This includes exposure to rheumatic diseases such as RA, polymyalgia rheumatica, and paraneoplastic syndromes [15]. Before joining the program, students and residents typically undergo a month of training in rural family medicine at other facilities. While students primarily observe under the guidance of medical teachers, residents have the autonomy to assess patients independently but are required to consult before prescribing medications or ordering diagnostic tests [16].

Participants

The research participants included family medicine trainees from the Family Medicine Department at Unnan City Hospital. Three medical teachers specializing in family medicine were affiliated with the department during the study period. Before their roles at Unnan City Hospital, the residents had undergone rural family medicine education at medical universities and tertiary hospitals. Additionally, they completed a year of family medicine training at rural hospitals, working alongside medical teachers and fellow residents. One resident began teaching within the curriculum in 2018 and 2019, followed by three more in 2020, 2021, and 2022. By 2022, 2023, and 2024, the department consisted of 13 physicians, including three family physicians and nine family medicine residents [17]. In total, 10 participants consented to participate in this research. 

Educational context regarding family medicine and rheumatology

The department trains medical residents using an educational curriculum based on the Japanese Primary Care Association's Board of Family Medicine, aligned with the World Standard of Education for Family Medicine. Three teachers support the hospital’s family medicine residency program. Throughout the program, residents are exposed to various clinical scenarios while treating patients. In their first year, residents work at Unnan City Hospital, managing common diseases in inpatient and outpatient settings. The following year, they spent six months at a rural clinic (Kakeya Clinic) to gain experience in home care and community-oriented primary care. The residents also complete an 18-month rotation at a general or community teaching hospital to expand their expertise in internal medicine, pediatrics, and emergency medicine. Each clinical setting is supervised by a medical teacher [17]. The curriculum incorporates near-peer learning and shared reading to enhance psychological safety in teaching family medicine and rheumatology [18-21].

Regarding rheumatology education, one of the family medicine educators (RO) conducted both on-the-job and off-the-job training for family medicine residents. RO completed a master's course in rheumatology at the University of South Wales and specializes in family medicine, medical education, and rheumatology at Unnan City Hospital. Within the hospital, the Department of Family Medicine manages patients suspected of having rheumatic diseases such as RA and vasculitis. Family medicine residents initially assess these patients and then consult with RO to discuss the diagnosis and management strategies. These discussions take place in both outpatient and inpatient settings, depending on the patient's condition.

Discussion and bedside teaching regarding rheumatic patients

Every weekday morning from 7:30 to 8:30, the Department of Family Medicine conducts regular team conferences to review and discuss patients whose conditions are worsening or of particular concern to the chief physicians [21]. During these conferences, family medicine residents present cases of rheumatic patients whose conditions are deteriorating or are challenging to diagnose. The residents engage in discussions with family medicine educators, including RO, to analyze these cases. In their analysis, residents consider potential immunological abnormalities in their patients and how to identify them through clinical histories, physical examinations, and specific diagnostic tests guided by medical educators. The educators facilitate these reviews and discussions, ultimately suggesting possible management strategies from both practical and educational perspectives.

Following the regular conference, family medicine educators and residents also visit the inpatients' bedsides to conduct joint examinations [22]. This hands-on approach allows residents to deepen their understanding of managing rheumatic diseases through direct examination and bedside discussions. In outpatient settings, family medicine residents request the presence of educators during patient encounters, following a process similar to that of inpatient cases to enhance learning. This study specifically focused on the discussion of patients with arthritis, particularly those diagnosed with RA and polymyalgia rheumatica.

Data collection

Data was collected by the primary researcher, who was also embedded in the setting as a family medicine educator (RO). This dual role as researcher and practitioner offered a distinctive perspective, allowing for deeper access to genuine interactions and experiences. Data was collected through direct participation, observation, reflective field notes, and one-on-one semi-structured interviews [11,12].

Observations

RO actively participated in morning team conferences, engaging daily in discussions regarding the cases presented by family medicine residents. During these conferences, as well as during clinical rounds and bedside discussions, RO focused on rheumatic diseases, particularly cases involving patients with systemic joint pain. Throughout this process, RO observed the content of the discussions and the interactions among the participants. Additionally, RO monitored their collaborative efforts in patient care within both outpatient and inpatient settings at the hospital. The observations were systematically focused on the residents' approaches to rheumatic diseases, specifically assessing their understanding of RA and polymyalgia rheumatica, their decision-making processes, and their management of patients [11,12].

Reflective field notes

RO kept detailed notes on personal reflections, interactions, and significant incidents that illuminated the dynamics of discussions and dialogues about RA and polymyalgia rheumatica with family medicine residents in both outpatient and inpatient settings. For instance, RO took notes while listening to conversations between family medicine teachers and residents concerning rheumatic diseases and inflammation. Due to concerns about patient and family privacy, these discussions were not recorded. Instead, RO focused on documenting how the participants approached rheumatic diseases and inflammatory conditions and any changes observed in their clinical practice [11,12].

Informal conversations

Casual, unstructured discussions with participants were conducted to gather spontaneous insights and perspectives on managing RA and polymyalgia rheumatica. These discussions occurred when RO had questions about the participants' understanding of rheumatology or when participants sought advice on managing their patients. RO aimed to explore how the participants perceived their rheumatic patients and to learn from their open-minded approach to managing rheumatic diseases [11,12].

One-on-one interview

RO conducted monthly one-on-one interviews with the participants to explore their learning experiences related to inflammatory diseases, including managing patients with arthritis, RA, and polymyalgia rheumatica. Each interview session lasted 30 minutes and took place in a conference room. During these interviews, RO reviewed relevant documents, such as meeting agendas, minutes, and training materials used in previous discussions and dialogues with the participants.

Data analysis

This study employed an autoethnographic approach [11,12]. After reviewing the field notes, conducting semi-structured in-depth interviews, and engaging in discussions with the research participants, RO coded the content and developed codebooks based on repeated reviews of the field notes to ensure reliability during the initial coding phase. The analysis primarily utilized process and concept coding methods [11]. Following this, RO iteratively refined the coding by inducing, merging, deleting, and revising codes, creating concepts by continually revisiting the research data and initial coding for the second phase. This phase focused on grouping tentative concepts and forming preliminary themes, with codes, ideas, and themes further refined through this iterative process. RO and the research participants engaged in ongoing discussions about the emerging concepts and themes throughout the study to ensure triangulation. The content of each interview was analyzed iteratively after each semi-structured interview. Ultimately, RO and CS reviewed the findings and agreed on the final themes.

Reflexivity

The results of this study were co-created through interactions between the researchers and participants. The research team brought together diverse expertise and perspectives on rural medical education. RO, a family physician and medical educator, holds a master's degree in medical education and family medicine and has extensive experience in working, teaching, and conducting research in rural settings. CS, a medical educator and professor at a medical university, has a medical education background specializing in community healthcare management and education. To minimize biases, the research team carefully discussed the findings from individual data analyses, considering alternative viewpoints and thoroughly exploring the meaning of the data.

Ethical consideration

The Unnan City Hospital Clinical Ethics Committee approved the study protocol (no.20240009).

## Results

Through this autoethnography, three themes were developed regarding learning inflammatory conditions through the experience-based learning of rheumatic diseases: comprehending arthritis as a systemic disease, managing dynamic conditions with prudence, and providing comprehensive and continuous care amid uncertainty. The participants initially viewed arthritis as a localized issue but learned to see it as part of systemic inflammation, enhancing holistic patient care. They recognized the fluctuating nature of autoimmune diseases and the importance of cautiously pursuing definitive diagnoses while avoiding premature treatments. Participants understood the potential harm from early use of steroids and immunosuppressants and emphasized preventive measures. They appreciated the need for persistent follow-up and confronting uncertainty in patient management. These insights underscore the importance of comprehensive education for family medicine residents in managing RA and other inflammatory diseases in rural settings (Table 1).

**Table 1:** Themes and concepts regarding learning autoimmunity from rheumatic diseases

Themes	Concepts
Comprehending Arthritis as a Systemic Disease	Recognizing Arthritis as Part of Systemic Inflammation
	Conceiving Systemic Diseases from Arthritis
	Maintaining Vigilance for Infections
Managing Dynamic Conditions with Prudence	Balancing the Quest for Definitive Diagnosis
	Understanding the Fluctuating Nature of Diseases
	Embracing Patience in Treatment Initiation
	Recognizing the Harm Potential of Treatments
Providing Comprehensive and Continuous Care Amid Uncertainty	Confronting Uncertainty
	Assessing Treatment Necessity Based on Symptom Progression
	Enhancing Preventive Awareness
	Persistently Following Up

**Figure 1:**
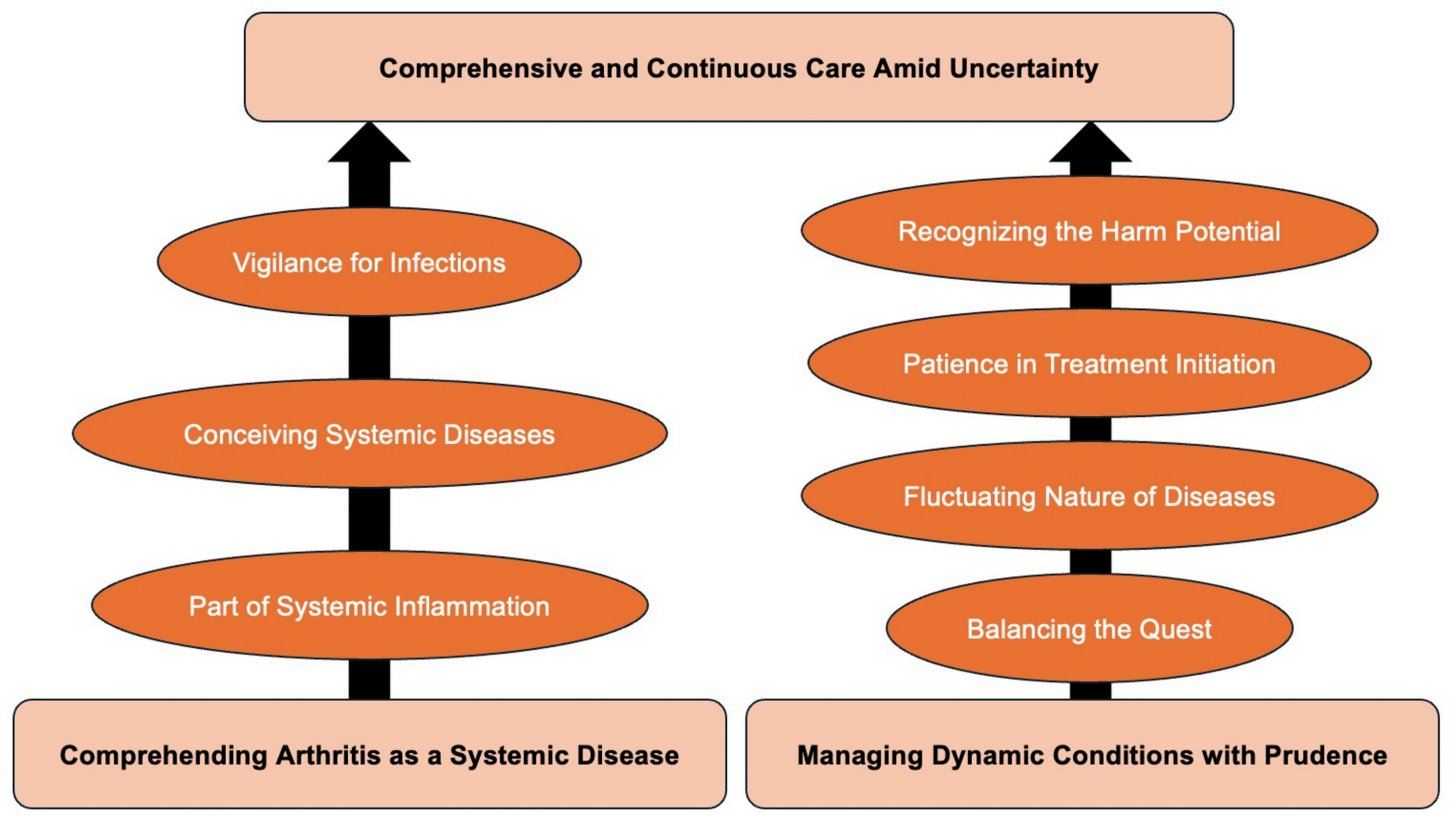
The conceptual figure of the learning of inflammatory conditions through the experience-based learning of rheumatic diseases Image credit: Ryuichi Ohta

The conceptual figure is shown in Figure 1.

Comprehending arthritis as a systemic disease

Recognizing Arthritis as Part of Systemic Inflammation 

Initially, participants viewed arthritis primarily as a localized symptom. One participant reflected, "At first, I thought of arthritis just in terms of the joint pain my patients were experiencing. It seemed like a standalone issue" (Participant 6). However, through dialogues with mentors, this perspective shifted. A participant noted, "My mentor explained that arthritis is not just about the joints; it indicates systemic inflammation. That conversation was eye-opening" (Participant 5). This realization led to an enhanced practice of viewing patients holistically. "I began to see my patients differently," another participant shared. "I realized that their joint pain was part of a larger inflammatory process affecting their whole body" (Participant 2). Considering the presence of joint pains and systemic inflammation comprehensively facilitated the residents to evaluate patients with arthritis systematically and look at systemic symptoms and changes in patients.

Conceiving Systemic Diseases From Arthritis 

Participants repeatedly managing patients with arthritis began to recognize that arthritis symptoms could indicate underlying systemic inflammation affecting various organs. One participant mentioned, "After seeing several patients with arthritis, I realized it wasn't just about joint pain. It was a sign of something bigger going on in the body" (Participant 1). This broadened their perspective significantly. "I started to think beyond the joints," Participant 3 shared. "I began considering how systemic inflammation could impact other organs, which changed how I approached treatment" (Participant 9). This shift allowed them to consider the impact on multiple organs, thus deepening their management approach. "Understanding the systemic nature of arthritis made me more thorough in my assessments and treatments," Participant 4 reflected, "ensuring I addressed all possible areas affected by inflammation." Focusing on systemic inflammation from arthritis provided various opportunities for medical residents to learn about the comprehensive management of inflammatory diseases and inquire about various possibilities for patients’ pathophysiology.

Maintaining Vigilance for Infections 

Participants learned the importance of consistently considering the potential for infections when diagnosing arthritis. One participant recalled, "In the beginning, I was mostly looking at collagen diseases when patients came in with joint pain and systemic symptoms" (Participant 4). This initial focus on differential diagnoses shifted over time. "I started to realize that infections could also present with joint pain because I had experienced several cases of sepsis and infectious endocarditis in which many colleagues suspected polymyalgia rheumatica," Participant 10 noted. This understanding led them to prioritize ruling out infections first. "Now, whenever I see a patient with arthritis, I consider infections a possible cause. It's become a crucial step in my diagnostic process," Participant 6 explained. The participants experienced several cases of sepsis and abscesses with patients suspected of autoimmune diseases. Through discussions with teachers and real experiences, medical residents learned the importance of suspecting infection in the initial phase of diagnosing autoimmune diseases. 

Managing dynamic conditions with prudence

Balancing the Quest for Definitive Diagnosis 

Participants learned that RA and autoimmune diseases could present with varied symptoms, emphasizing the importance of pursuing a definitive diagnosis while continuously assessing for diverse possibilities. One participant noted, "Initially, I fixated on quickly finding a definitive diagnosis, but I realized autoimmune diseases are complex and varied." (Participant 7) Mentors highlighted the need for flexibility, as another participant shared, "We start with the most likely diagnosis and adjust as we gather more data. I could learn through the discussion with teachers and colleagues that I should not close my differential diagnosis process because autoimmunity can change in the process." (Participant 7) This approach involves thorough initial assessment, ongoing reassessment, and effective patient communication, ensuring optimal care despite diagnostic challenges. Medical residents learned that as definitive diagnosis is essential, they needed to consider the different diagnosis possibilities when encountering challenging symptoms in the follow-up of their autoimmune patients.

Understanding the Fluctuating Nature of Diseases 

Through their interactions with rheumatoid arthritis and polymyalgia rheumatica patients, participants recognized that symptoms could worsen or improve regardless of treatment adjustments. One participant noted, "I observed patients whose symptoms changed dramatically without any changes in their treatment" (Participant 1). They realized that autoimmune diseases frequently fluctuate. Another participant shared, "I learned that factors like seasons or changes in living environments can significantly impact the disease course" (Participant 7). This understanding emphasized the importance of continuous monitoring and flexibility in management. "We need to be adaptable in our approach, knowing that these diseases are inherently unpredictable," Participant 2 concluded. The participants realized that rheumatic diseases could be affected not only by their treatments but by various factors and that they should not assess patients’ conditions in the short term, demanding long-term visions for the management.

Embracing Patience in Treatment Initiation 

Participants initially felt compelled to start anti-inflammatory treatments, such as steroids, when patients presented with joint pain or unclear symptoms. One participant shared, "I used to think immediate treatment was necessary to relieve symptoms" (Participant 9). However, through experience and mentorship, they learned that premature treatment without a clear understanding of the pathology could complicate management. Another participant reflected, "Starting treatment too early could mask the true underlying condition and lead to more complex issues down the line" (Participant 8). This realization underscored the importance of avoiding hasty anti-inflammatory interventions until a definitive diagnosis was made. They came to appreciate that patience in treatment initiation often resulted in more accurate diagnoses and better long-term outcomes, ensuring a more effective and tailored patient care approach.

Recognizing the Harm Potential of Treatments 

Participants were initially prone to early use of steroids and immunosuppressants. One participant noted, "I often started patients on steroids right away, thinking it would quickly alleviate their symptoms" (Participant 3). However, experiencing various complications in patients and discussions with mentors highlighted the high risk of harm from such treatments. "Seeing patients develop serious side effects made me realize the potential dangers of these medications," Participant 1 shared. These experiences prompted them to choose treatment methods more cautiously. "I now weigh the benefits and risks more carefully and opt for less aggressive treatments, when possible," Participant 7 explained. This cautious approach led to safer, more effective patient care. Through various experiences and discussions about autoimmune diseases, the participants realized that rheumatic medicine always had negative impacts on patients’ physiology and that the balance between advantages and disadvantages should be considered strenuously. 

Providing comprehensive and continuous care amid uncertainty

Confronting Uncertainty 

Participants realized the inherent uncertainty in managing autoimmune disease patients, noting instances where diagnoses changed during follow-ups or patients improved without treatment changes. One participant shared, "There were times when a patient's condition improved spontaneously, or their diagnosis evolved" (Participant 10). They learned the importance of following symptoms closely. "It became clear that continuous monitoring and reassessment were crucial," another participant noted (Participant 7). Facing this uncertainty, they developed a more flexible approach to patient care. "We had to be comfortable with not having all the answers immediately and focus on thorough, ongoing evaluation," Participant 5 explained. This approach ensured they could adapt to changing conditions and provide the best care. The participants learned that a definite diagnosis was essential. Still, continuous changes in patient conditions should be considered at any time, and patients should be flexible regarding their symptoms.

Assessing Treatment Necessity Based on Symptom Progression 

Participants appreciated the significance of monitoring the progression speed of symptoms. One participant noted, "I learned that not all symptoms require immediate intervention, especially if they're progressing slowly" (Participant 2). They recognized the importance of careful observation for slowly progressing conditions or cases that improved naturally. Another participant shared, "In some cases, symptoms would improve on their own, and rushing to lower immune responses with medication could do more harm than good" (Participant 5). This understanding led them to adopt a more measured approach. "We realized that sometimes, the best course of action is to wait and watch, ensuring that we don't intervene unnecessarily," Participant 1 explained. This approach helped avoid premature treatments and promoted better long-term patient outcomes. The participants learned that medical treatments were not the only way to approach the problem, and careful observation was vital for effective treatments of autoimmune diseases.

Enhancing Preventive Awareness 

While treating patients with RA and polymyalgia rheumatica, participants became acutely aware of the importance of preventing complications arising during the treatment process. One participant shared, "I realized that our treatments could sometimes lead to significant complications if not managed carefully" (Participant 6). Discussions with mentors underscored the need for a comprehensive view of patients with inflammatory diseases. "My mentor emphasized looking at the whole patient, not just the immediate symptoms," another participant noted (Participant 1). This holistic approach led them to implement preventive measures more effectively. "We started focusing on preventing issues before they arose, such as monitoring for potential side effects such as hyperglycemia and osteoporosis and addressing them early. I could also learn the importance of family physicians’ management of autoimmune disease in community hospitals," Participant 10 explained. This proactive strategy improved overall patient care and outcomes. The participants realized that comprehensive management was essential for autoimmune diseases, enhanced by family physicians in community hospitals.

Persistently Following Up 

Participants realized the challenges of regularly following up with autoimmune disease patients. One participant noted, "It was difficult to keep up with the frequent follow-ups, but it was necessary" (Participant 9). They valued the importance of persistently addressing patients' recurring pain symptoms. "Patients often have ongoing issues, and consistent follow-up is crucial to manage these effectively," Participant 4 shared. Maintaining a continuous approach to their care became a priority. "We learned that regular check-ins and adjustments to treatment plans were key to providing the best care," Participant 1 explained. This persistent follow-up ensured that patients received the needed attention and improved their long-term outcomes. Through persistent follow-ups with medical educators, the participants learned that family physicians dealing with autoimmune diseases need to approach multiple and fluctuating problems comprehensively to stabilize their symptoms.

## Discussion

Our study aimed to clarify the educational process and learning tips for family physicians in managing RA, polymyalgia rheumatica, and other autoimmune diseases within rural hospital settings, focusing on enhancing the practical education and experience-based learning of family medicine residents. With autoethnography, we uncovered significant insights into how family medicine residents comprehend and manage RA and related rheumatic conditions in rural contexts through the education by family physicians with a particular interest in rheumatology.

Comprehending arthritis as a systemic disease can be essential for managing autoimmune diseases in family medicine. Our findings indicate that family medicine residents initially view arthritis as a localized condition. However, through experiential learning and mentorship, they understand arthritis as a systemic inflammatory disease. This transition is critical as it shifts the focus from merely addressing joint pain to considering the broader systemic implications, thereby enhancing holistic patient care [23]. Previous studies corroborate this finding, emphasizing the need for comprehensive education that integrates the systemic nature of inflammatory diseases into primary care training [23,24]. As family physicians, family medicine residents are demanded to deal with various medical issues of patients comprehensively [25]. As shown in this study, they can learn about autoimmune diseases, including RA and polymyalgia rheumatica, like other chronic diseases, and deal with them comprehensively as one of their patient’s chronic diseases.

In family medicine, managing dynamic conditions with prudence is essential for autoimmune disease care and should be acquired through continual training. In this research, participants learned to balance the pursuit of a definitive diagnosis with the necessity for ongoing assessment and flexibility. This learning process is essential in managing autoimmune diseases, which often present with varied and fluctuating symptoms [26]. The emphasis on avoiding premature treatments aligns with the findings of previous research, which advocate for a cautious approach to initiating aggressive therapies such as steroids and immunosuppressants to prevent potential harm [27]. This prudence in treatment initiation and management highlights the importance of developing diagnostic acumen and clinical judgment among family medicine residents. As system-specific specialists, family physicians should meticulously be cautious about their patients’ changes in symptoms and adjust their treatments effectively through dialogues with patients.

For the effective care of rheumatic diseases, family physicians can acquire the skills and attitudes to provide comprehensive and continuous care amid Uncertainty. This study revealed the challenges of managing RA amid diagnostic uncertainty and the need for persistent follow-up. Family medicine residents learned the value of continuous monitoring and adapting treatment plans based on symptom progression. This approach aligns with the recommendations from previous research, emphasizing the importance of continuous care and monitoring in managing chronic inflammatory conditions [28]. Managing uncertainty and providing comprehensive care is crucial in rural settings where access to rheumatologists is limited. Especially in the present VUCA (Volatility, Uncertainty, Complexity, and Ambiguity) world, rural family physicians can learn how to deal with uncertainty in complex and chaotic cases [29,30]. Rheumatic diseases can complicate patients’ situations as well. Rural family physicians should learn about rheumatic diseases effectively through the improvement of the education of rheumatology.

Our study has several limitations. Firstly, while providing deep contextual understanding, the researcher's subjective experiences and perspectives may influence the autoethnographic approach. This could lead to potential biases in interpreting the data. Future research could benefit from a mixed-methods approach, incorporating quantitative data to validate and complement the qualitative findings. Secondly, the study was conducted in a single rural hospital, which may limit the generalizability of the results to other settings. Rural hospitals vary significantly regarding resources, patient populations, and educational structures. Expanding the study to include multiple rural hospitals would provide a broader understanding of the educational processes and challenges in different contexts. Thirdly, relying on self-reported data from participants, including reflective field notes and interviews, may introduce recall and social desirability biases. Participants might have provided responses that they believed were expected rather than their genuine experiences. Triangulating these self-reported data with objective measures, such as patient outcomes and performance assessments, could strengthen the validity of the findings.

## Conclusions

Our study underscores the critical need for comprehensive and experiential learning in managing RA and other inflammatory diseases among family medicine residents in rural settings. The findings highlight the importance of viewing arthritis as a systemic disease, the prudence required to manage dynamic conditions, and the necessity to provide continuous care amid diagnostic uncertainty. These insights can inform the development of targeted educational programs and curricula for family medicine residents, ultimately improving patient care in rural areas. Future research should focus on expanding the study to multiple rural settings, incorporating quantitative data, and exploring the long-term impact of enhanced education on patient outcomes. By addressing these areas, we can further refine the educational process for family physicians and improve the quality of care for patients with rheumatic diseases in rural communities.

## References

[1] Perniola S, Chimenti MS, Spinelli FR (2023). Rheumatoid arthritis from easy to complex disease: from the "2022 GISEA International Symposium". J Clin Med.

[2] Inchingolo F, Inchingolo AM, Fatone MC (2024). Management of rheumatoid arthritis in primary care: a scoping review. Int J Environ Res Public Health.

[3] Rangiah S, Govender I, Badat Z (2020). A primary care approach to the management of arthritis. S Afr Fam Pract (2004).

[4] Ohta R, Sano C (2023). Integrating clinical and socio-environmental approaches in managing rheumatoid arthritis with social determinants of health: a case study of an elderly patient in rural Japan. Cureus.

[5] Ohta R, Sano C (2024). Factors affecting the duration of initial medical care seeking among older rural patients diagnosed with rheumatoid arthritis: a retrospective cohort study. BMC Rheumatol.

[6] Lagacé S, Julien AS, Rheault C, Bessette L, Michou L (2020). What are the rheumatology educational preferences of family medicine residents? A descriptive study. Clin Rheumatol.

[7] Hosie GA (2000). Teaching rheumatology in primary care. Ann Rheum Dis.

[8] Siani M, Dubovi I, Borushko A, Haskel-Ittah M (2024). Teaching immunology in the 21st century: a scoping review of emerging challenges and strategies. Int J Sci Educ.

[9] Pianarosa E, Chomistek K, Hsiao R, Anwar S, Umaefulam V, Hazlewood G, Barnabe C (2022). Global rural and remote patients with rheumatoid arthritis: a systematic review. Arthritis Care Res (Hoboken).

[10] Hollick RJ, Macfarlane GJ (2021). Association of rural setting with poorer disease outcomes for patients with rheumatic diseases: results from a systematic review of the literature. Arthritis Care Res (Hoboken).

[11] Poerwandari EK (2021). Minimizing bias and maximizing the potential strengths of autoethnography as a narrative research. Japan Psychol Res.

[12] Bochner AP, Ellis C (2022). Why autoethnography?. Soc Work Soc Sci Rev.

[13] Amano S, Ohta R, Sano C (2024). Relationship between anemia and readmission among older patients in rural community hospitals: a retrospective cohort study. J Clin Med.

[14] Ohta R, Nitta T, Shimizu A, Sano C (2024). Role of family medicine physicians in providing nutrition support to older patients admitted to orthopedics departments: a grounded theory approach. BMC Prim Care.

[15] Ohta R, Ryu Y, Katsube T, Otani J, Moriwaki Y (2021). Strengths and challenges for medical students and residents in rural Japan. Fam Med.

[16] Nishikura N, Ohta R, Sano C (2021). Effect of residents-as-teachers in rural community-based medical education on the learning of medical students and residents: a thematic analysis. Int J Environ Res Public Health.

[17] Ohta R, Ryu Y, Sano C (2021). Family medicine education at a rural hospital in Japan: impact on institution and trainees. Int J Environ Res Public Health.

[18] Nishikura N, Ohta R, Sano C (2023). Implementation of near-peer learning for the sustainability of rural family medicine education. Cureus.

[19] Ohta R, Katsube T, Sano C (2023). Shared reading as a community of practice for overcoming the generation gap and improving psychological safety in rural family medicine education: a grounded-theory approach. Cureus.

[20] Ohta R, Katsube T, Sano C (2023). Psychological safety and self-regulated learning through near-peer learning for the sustainability of rural community-based medical education: grounded theory approach. Rural Remote Health.

[21] Ohta R, Sano C (2024). The quality of family medicine team conferences through the lens of a director: an autoethnography. Cureus.

[22] Ohta R, Sano C (2022). Bedside teaching in rural family medicine education in Japan. Int J Environ Res Public Health.

[23] Moutsopoulos HM (2021). Autoimmune rheumatic diseases: one or many diseases?. J Transl Autoimmun.

[24] Robbins RC, Maciuba JM, Maggio LA, Samuel A (2023). Continuing professional development in rheumatology for primary care clinicians: a systematic review. Arthritis Care Res (Hoboken).

[25] Randall E, Grudniewicz A, Rudoler D, Goldsmith L, Lavergne R (2023). How do family medicine residents and early career family physicians talk about comprehensiveness in primary care?. Ann Fam Med.

[26] van Onna M, Boonen A (2022). Challenges in the management of older patients with inflammatory rheumatic diseases. Nat Rev Rheumatol.

[27] Guo Q, Wang Y, Xu D, Nossent J, Pavlos NJ, Xu J (2018). Rheumatoid arthritis: pathological mechanisms and modern pharmacologic therapies. Bone Res.

[28] Knight JC, Gadag V, Worrall G, Sikdar K (2007). Continuity of family physician care - is it more important for older people and the chronically-ill?. Ann Epidemiol.

[29] Ohta R, Sano C (2023). Case report-driven medical education in rural family medicine education: a thematic analysis. Healthcare (Basel).

[30] Maini A, Saravanan Y, Singh TA, Fyfe M (2020). Coaching skills for medical education in a VUCA world. Med Teach.

